# Treatment Strategy Research on a Squirrel-Cage Induction Motor with Broken Rotor Bar Faults

**DOI:** 10.3390/s22124345

**Published:** 2022-06-08

**Authors:** Yucai Wu, Shuqiong Sun, Qingfei An, Xu Lie

**Affiliations:** 1 Department of Electrical, North China Electric Power University, Yonghua Street No. 619, Baoding 071000, China; hsdlssq@163.com; 2 State Grid Shandong Power Company Laiwu Power Supply Company, Luzhongxi Street No. 21, Jinan 271100, China; anqingfei1994@163.com; 3 Department of Electronic and Electrical Engineering, University of Strathclyde, Glasgow G1 1XW, UK; lie.xu@strath.ac.uk

**Keywords:** squirrel-cage induction motor, broken rotor bar, loss, efficiency, fault simulation experiment

## Abstract

Squirrel-cage induction motors are increasingly displaying a broken rotor bar fault, which represents both a technical problem and an economic problem. After confirming that the broken rotor bars do not affect the normal start-up and basic working performance of the squirrel-cage induction motor, this paper focuses on the loss and efficiency changes of the motor brought about by the broken rotor bar fault. Using finite element simulation and experimentation, various losses like stator copper loss, iron loss, rotor copper loss, mechanical loss and additional losses, total loss and efficiency are obtained. By combining price and cost factors, the cost-effective measures that can be taken after the occurrence of different degrees of broken bars are evaluated here to provide guidance for correctly handling this problem.

## 1. Introduction

Squirrel-cage induction motors (IMs) have a simple structure, are convenient to manufacture, cost-effective and durable. These motors are widely used in various fields with power ranging from tens of watts to several megawatts. Due to harsh operating environments, frequent heavy-duty starting, improper manufacturing and maintenance, the rotor bars in squirrel-cage IMs are prone to fracture. Data show that the occurrence rate for broken rotor bar (BRB) faults accounts for about 10% of all failures in squirrel-cage IMs [1,2]. BRB faults lead to the deterioration of the squirrel-cage IM’s operating performance. Therefore, it is necessary to detect BRB faults quickly and accurately so as to formulate correction strategies to deal with the issue. This allows us to ensure safe and reliable motor operation.

A great deal of research on BRB fault diagnosis has already been carried out. Here, a great deal of emphasis has been placed on two aspects: field variation characteristics and fault diagnosis methods. In terms of field variation characteristics, variation laws play a role in various physical fields when a squirrel-cage IM has a BRB fault, including the electromagnetic field [3,4], electromagnetic force [5,6], thermal stress [7,8] and temperature field [9,10]. These are important for improving the formation mechanism of fault characteristics, predicting development trends and realizing an on-line diagnosis. Various fault diagnosis methods have been proposed based on characteristic quantities. These include such things as the stator current method [11,12,13,14,15,16], residual voltage method [17,18], instantaneous power method [19,20] and electromagnetic torque method [21,22]. So far when dealing with BRB faults in the squirrel-cage IM, the focus has usually been on fast and sensitive fault detection [23]. This has been accomplished by extracting effective fault characteristics, as well as other strategies to avoid the possible negative impacts of the fault.

As squirrel-cage IMs are usually small capacity and mostly used in the field of driving, they are not as critical as large-capacity synchronous generators. The BRB fault itself is not a fatal failure in squirrel-cage IMs; usually, they can continue operating under minor BRB faults. Therefore, in the process of studying these faults, the following questions should be considered: will one or two broken rotor bars prevent the unit from continuing to operate? What decisions should the equipment operator make after the unit suffers a BRB fault? It is necessary to accurately calculate and evaluate the relevant technical and economic data under the specific condition of the BRB fault. This allows operators to make correct decisions.

There is a great deal of literature about the loss of an induction motor under normal working conditions. For example, the paper [24] shows the relationship between the temperature rise of the stator winding and the speed of the induction motor under load. It obtains a method to evaluate the efficiency of the motor through short-term operation, which avoids the long-term thermal stability test. In [25], the core loss of an induction motor is calculated using the classical analytical model, and the accuracy of the analytical model is verified by comparing the analytical results with the experimental results. Here, the loss characteristics of different ferromagnetic materials are compared. An analytical model for a three-phase induction motor is proposed in the paper [26]. Here, the iron loss and copper loss of the motor are calculated, and the relationship between the power loss of the motor and its equivalent circuit parameters is obtained. To reduce interference in the operation, the efficiency change of an induction motor under different load levels is studied in the form of an equivalent circuit in paper [27]. Here, the calculation accuracy is exemplary. In [28], an equivalent electrical circuit for dual stator winding induction machines that considers the iron loss effect is presented, and its iron loss is estimated. Another paper [29] analyzed the increase in core eddy current loss caused by PWM voltage harmonic components. This paper deduces the influence of PWM switch parameters on eddy current loss, and it puts forward a detailed formula for iron loss prediction. In [30], the iron loss of an induction motor is studied by injecting high-order harmonics into a PMW inverter. Here, the relationship between the harmonic loss factor and the harmonic number is obtained. Papers [31,32,33] point out that in the dynamic process of an asynchronous motor, the given torque value set by the steady-state loss minimization scheme will increase the motor loss. A scheme of motor loss reduction considering the dynamic process is then proposed. Here, the motor flux is dynamically adjusted according to the flux demand within the dynamic process. In [34], by simulating a series of linear, incremental permeability, the cage losses in IMS, which are computed due to the harmonic fields considering the fundamental flux saturation, are calculated and a finite element analysis (FEA) procedure is proposed. The transient loss in an induction motor with rotor field-oriented control is studied in paper [35]. Here, the relationship between rotor d-axis flux and transient energy efficiency is derived, and a scheme to reduce the transient process energy loss through open-loop control is proposed.

At present, there has been little research on the efficiency of induction motors under fault conditions. In papers [36,37], based on the equivalent circuit of an induction motor, the efficiency characteristics under an unbalanced load supply are studied, and an efficiency prediction model is proposed. Paper [38] studies the influence of broken rotor bar faults on the efficiency of induction motors. It obtains the time fluctuation characteristics of motor efficiency under broken rotor bar faults through a finite element simulation and completes the fault simulation experiment. Paper [39] measured the efficiency of an asynchronous motor under different load levels through experimentation. It obtained the relationship between efficiency and the degree of fault in a broken bar rotor.

**Figure 1:**
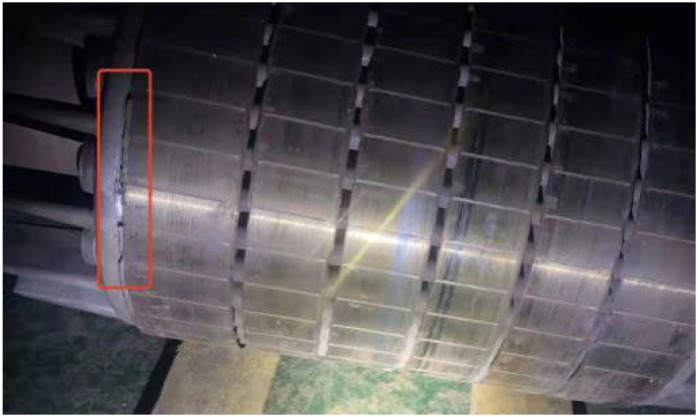
A faulty rotor.

Since the BRB fault has a significant impact on the efficiency of the asynchronous motor, the economy of continuous operation of an asynchronous motor after the BRB fault needs to be seriously considered, however, the actual situation is that professionals on the scene did not think about this problem rationally. For example, several rotor bars of an asynchronous motor in a factory in China were broken, as shown in Figure 1, but the operator insisted on continuing to use the rotor until it was completely unusable.

This article introduces three strategies for dealing with BRB faults of the squirrel-cage IM in order to save costs and improve operating efficiency. For this, the relationship between BRB faults and starting characteristics, running characteristics, losses and efficiency of squirrel-cage IM is studied, and a comparison is made between the extra electricity costs of a motor that continues to run after a BRB fault, with the economic cost of timely maintenance. The remainder of this article is structured as follows: Section 2 analyzes the effect of a BRB fault on starting and operating characteristics of a squirrel-cage IM and performs finite element simulation using a YKK3552-4 squirrel-cage IM as a model. Section 3 presents the computational model for the loss and efficiency of a squirrel-cage IM. The experimental results are discussed in Section 4. Section 5 provides strategies to deal with BRB faults from an economic point of view. Finally, Section 6 summarizes the research work of this paper and suggests future research directions.

## 2. Influence BRB Faults on Starting and Operating Characteristics

For squirrel-cage IMs with broken rotor bars, starting performance immediately deteriorates. The main parameters characterizing the starting performance are the starting torque and starting time. During unit start up, the starting torque has to be greater than the load torque, while the starting time is an indirect reflection of the starting torque. The smaller the starting torque is, the longer the starting time that is required. However, as long as the motor can be started normally following a BRB fault, it is theoretically possible to use this. That said, its running performance may be still affected to an extent.

In this paper, a YKK3552-4 squirrel-cage IM produced by Xiangtan Electric Machinery Factory in China is selected for investigation and its starting performance is analyzed using a finite element simulation. The parameters of the motor are shown in Table 1.

Neglecting the influence of the axial magnetic field of the squirrel-cage IM and the eddy current effect of the stator winding and the iron core, this field-circuit coupling two-dimensional transient joint simulation model of the squirrel-cage IM was built by Ansoft Maxwell and Ansoft Simplorer software.

**Figure 2:**
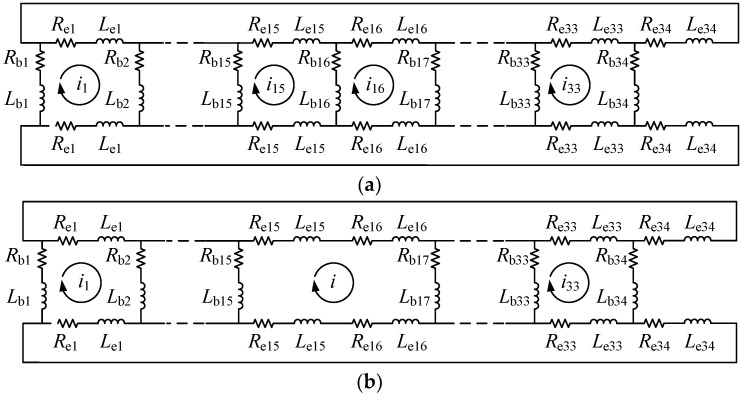
Model of circuit with BRB fault: (**a**) normal rotor; (**b**) broken #16 bar.

Figure 2a,b are the equivalent circuits with normal rotor bars and a broken #16 bar, respectively, and the situation is similar when multiple bars are broken.

In Figure 2, *R*_e_ and *L*_e_ are the respective end ring resistance and the inductance, *R*_b_ and *L*_b_ are the respective conductor resistance and inductance, and *i* is the rotor loop current.

It becomes apparent that the BRB fault causes an asymmetry in the rotor circuit, and the mesh current structure of the rotor also changes. In addition, the magnetic field generated by the current also becomes asymmetrical.

**Figure 3:**
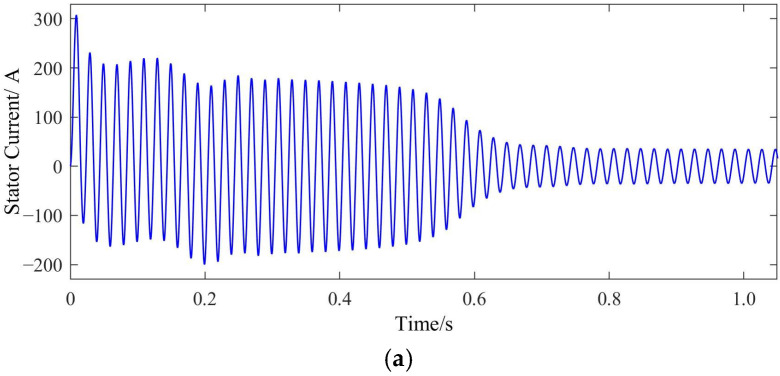

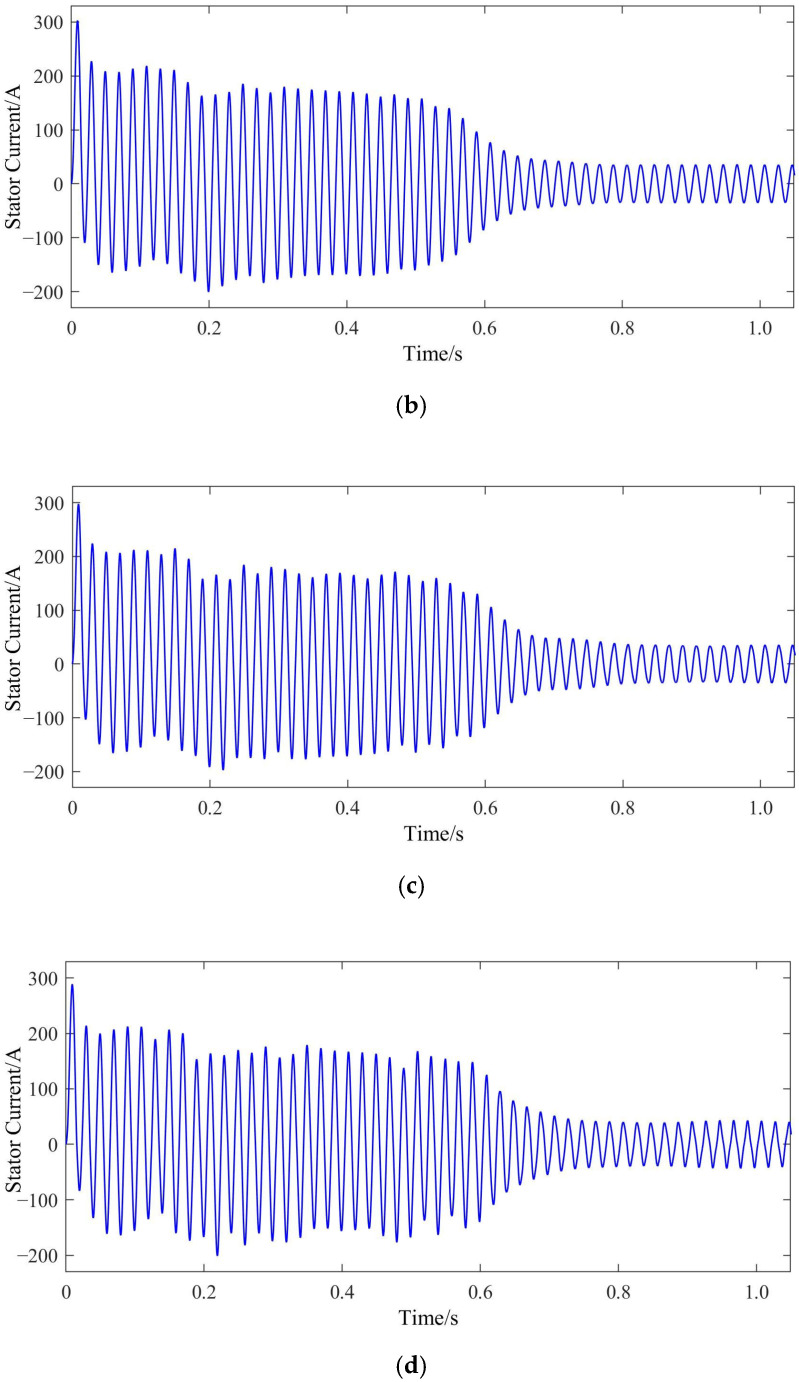
Current waveforms at different fault levels:(**a**) normal; (**b**) one broken bar; (**c**) Two broken bars; (**d**) Three broken bars.

**Figure 4:**
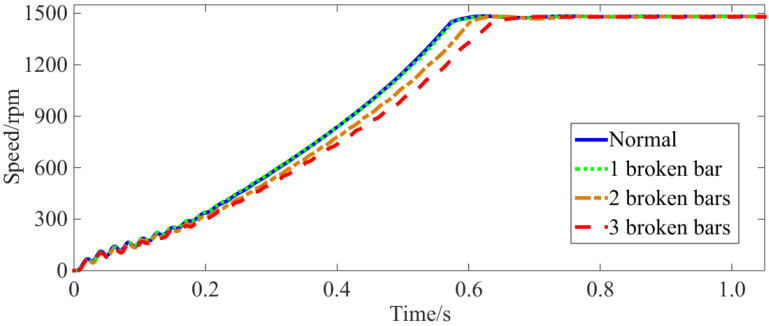
Speed waveforms at different fault levels.

In the model, a constant rated-load torque is applied to the squirrel-cage IM, and the modes of the normal rotor bar, one broken bar, two broken bars and three broken bars are set, respectively. Finite element joint simulation covering the starting process is then carried out. The resulting motor starting current waveform (taking phase A as an example) and the rotation speed waveform are shown in Figure 3 and Figure 4, respectively.

From Figure 3 and Figure 4, it becomes apparent that under different rotor bar conditions, the squirrel-cage IMs can still start normally with only small changes in the current waveforms. This shows that the BRB fault has not seriously affected the starting process, especially in the case of one broken bar failure. Here, the starting time is almost exactly the same as that with a normal rotor bar. The starting time is prolonged by 5% in the case of two broken bars and 10% with three broken bars. The start up of the motor is a short-term process with a total duration of only about 0.7 s, and the increased time caused by broken bars is negligible compared to the long-term steady-state operation process. At the end of the starting process, the speed of the motor becomes very stable with no noticeable fluctuations. This indicates that the steady-state mechanical power output of the motor experiences no obvious changes. Therefore, under these broken bar conditions, the squirrel-cage IM technically meets all requirements for continuous operation.

**Figure 5:**
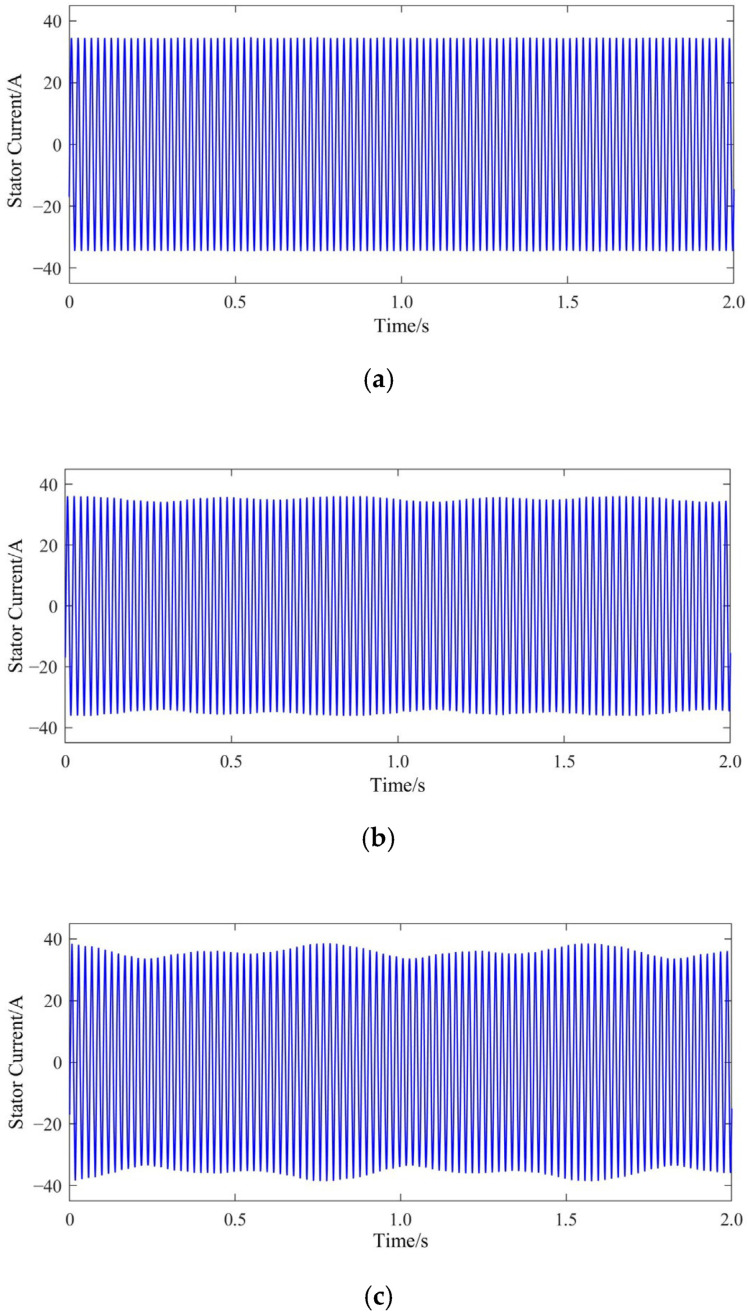

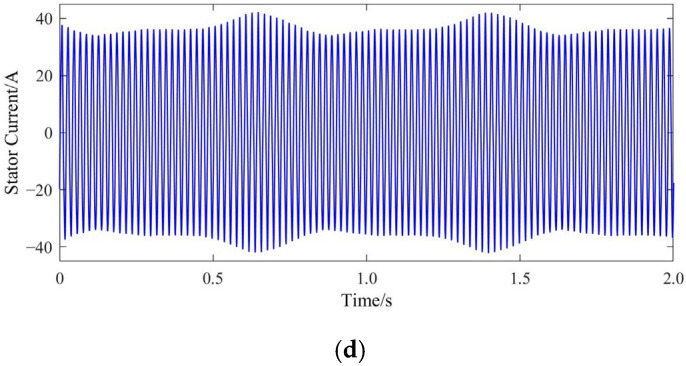
Stator phase A current: (**a**) normal; (**b**) one broken bar; (**c**) two broken bars; (**d**) three broken bars.

**Figure 6:**
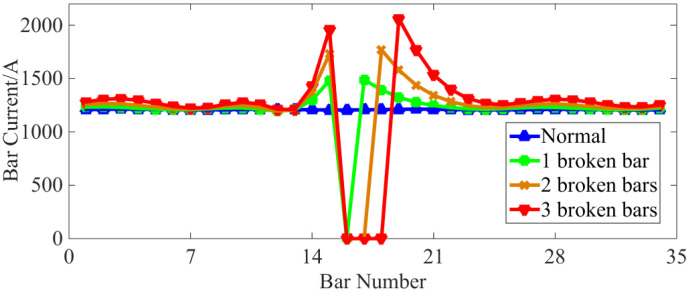
Current change in each bar.

**Figure 7:**
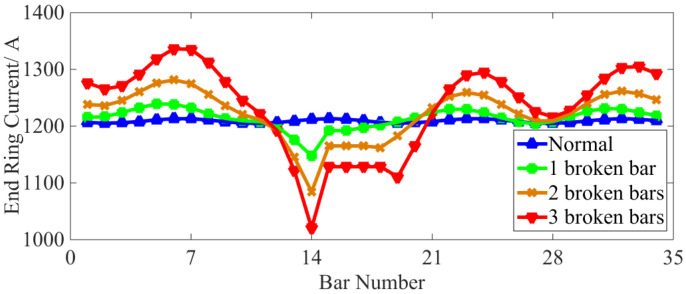
Current change in each end ring.

Further investigations into the stator winding steady-state current (taking phase A as an example), the current of each bar and the end ring (RMS value) are displayed in Figure 5, Figure 6 and Figure 7, respectively.

As can be seen from Figure 5, the amplitude of the stator current fluctuates when the rotor has broken bars. The larger the number of broken bars in the rotor, the greater the amplitude of the fluctuation of the stator current amplitude, and the higher the amplitude of the current.

As can be seen from Figure 6, the current of the broken bar becomes zero, while the current amplitude of the bars adjacent to the broken bar increases significantly. Concurrently, the current amplitude of the bars farther away from the failed bar only sees small increases. This phenomenon indicates that the rotor compensates for the magnetic field asymmetry caused by the broken bars by increasing the bar current near the fault position in order to improve the magnetic field in the squirrel-cage IM. When affected by the BRB fault, the current amplitude of each bar of the whole squirrel-cage rotor shows a slight distribution fluctuation in space. The more broken rotor bars there are, the more severe the above phenomenon becomes.

As can be seen from Figure 7, when a rotor bar is broken, the current of two segments of the end ring adjacent to the broken bar remains the same, and the current values are obviously reduced. At the same time, the amplitudes of the end ring current farthest away from the broken bar are significantly increased and experience higher fluctuations. With an increased number of BRBs, the fluctuations in the current amplitude of each end ring also increase.

## 3. Loss and Efficiency Calculation

Power loss in three-phase squirrel-cage IMs mainly consists of the following five parts: stator copper loss, iron loss, rotor copper loss, mechanical loss and additional loss [40]. Among these, stator copper loss, iron loss and rotor copper loss belong to the electromagnetic loss category and can be calculated using finite element simulation. Mechanical loss and additional loss can be estimated using a traditional empirical formula. The following calculation models are, respectively, established for each loss.

### 3.1. Stator Copper Loss

**Figure 8:**
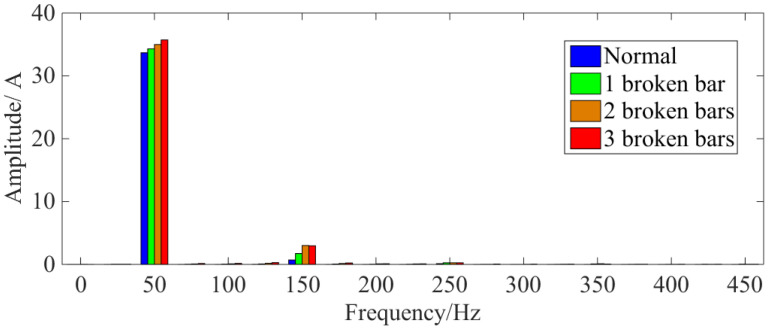
FFT results of stator current signal under rated load.

When the rotor bars break, squirrel-cage IMs increase their stator current in order to maintain constant torque output. Figure 8 shows the FFT results of the stator current for the squirrel-cage IM. Here, it can be seen that the fundamental stator current increases after the BRB fault occurs.

In addition, the stator winding also induces small harmonic magnetic fields [41,42] at frequencies of (1 ± 2 *ks*)*f*_1_ (where *s* is the slip, *f*_1_ is the power supply frequency and *k* is a positive integer) and [*λ*(1 − *s*)/*p* ± *s*]*f*_1_ (where *p* is the number of motor pole-pairs, *λ* = 1, 3, 5, …, 2*k* − 1). Due to the interference of saturation and other factors, even when the squirrel-cage IM is supplied with sinusoidal voltage, the stator current still contains odd harmonics, especially the 5th and 7th [43]. In addition, because the stator windings are mostly in delta connections, there will also be a 3rd harmonic circulating current inside.

Considering the above factors, the stator copper loss should be calculated using phase current instead of line current. It should also not be limited to a single stator period (0.02 s). Instead, the fluctuations in Figure 5 should be considered by taking the RMS value of multiple periods. Thus, stator copper loss is calculated as:

(1) $$p_{\mathrm{sCu}}=\frac{1}{nT}\int_{0}^{nT}R_{p}\left(i_{A}^{2}+i_{B}^{2}+i_{C}^{2}\right)dt$$

where *T* is the stator current period, *n* is the number of stator current periods, *R*_p_ is the stator phase resistance and *i*_A_, *i*_B_, and *i*_C_ are the time-domain current waveforms of phase A, B, and C, respectively.

**Figure 9:**
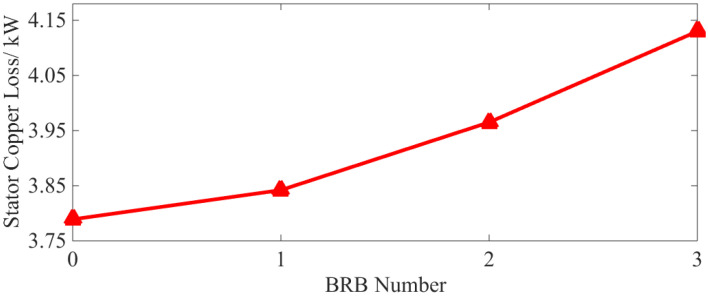
Characteristic curve of stator copper loss.

After substituting the steady-state three-phase current in Figure 5 into Equation (1) and setting *n* = 100, i.e., sampling time of 2 s, the characteristic curve of stator copper loss is provided in Figure 9.

Figure 9 shows that the stator copper loss of the squirrel-cage IM increases after the rotor bar has been broken. Additionally, the larger the number of broken bars, the higher the stator copper loss and the growth rate of copper loss. This phenomenon shows that the increase in the fundamental wave and the “useless” harmonic current in the stator winding promotes an increase in the stator copper loss.

### 3.2. Rotor Copper Loss

Due to the relative motion that occurs between the stator harmonic magnetic field and the rotor, a high-frequency harmonic current appears in the rotor bars. Here, the skin effect will cause an uneven distribution of the bar current [44]. The rotor bars are divided into small grid units in the finite element method, and the current in each unit is approximated to be evenly distributed. Then the total copper loss of the rotor bars can be obtained by adding the copper loss in each grid as:

(2) $$p_{\mathrm{rCu}1}=\sum_{i}\frac{1}{\sigma }L_{b}S_{i}J_{i}^{2}$$

where *σ* is the conductivity of the bar, *L*_b_ is the effective length of the bar, *S_i_* is the area of the *i*-th bar unit and *J_i_* is the RMS value of the current density.

In the external circuit of the finite element joint simulation model, the effect of the rotor end ring is simulated by setting the end ring resistance and leakage inductance between adjacent bars. The total copper loss of the rotor end ring is calculated as:

(3) $$p_{\mathrm{rCu}2}=\sum_{j=1}^{2Z_{2}}I_{ej}^{2}\cdot R_{ej}$$

where *Z*_2_ is the number of rotor bars, *I*_e*j*_ is the RMS value of the *j*-th end ring current, *R*_e*j*_ is *j*-th end ring resistance.

**Figure 10:**
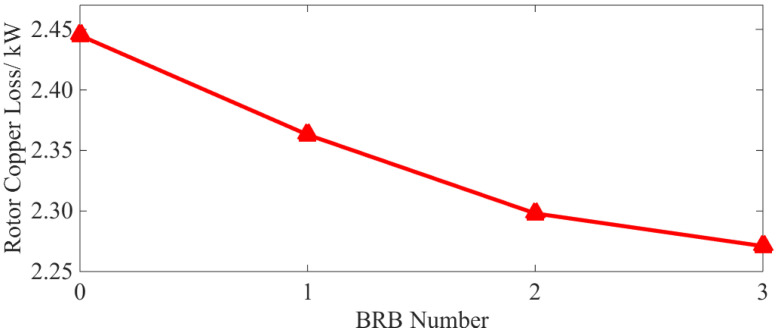
Characteristic curve of rotor copper loss.

According to Equations (2) and (3), the loss of rotor bars and end rings can be calculated, respectively, to obtain the total rotor copper loss. This is shown in Figure 10.

As can be observed from Figure 10, the total rotor copper loss decreases when a BRB fault occurs, and the more broken rotor bars there are, the lower the total copper loss. At the same time, in Figure 6, it can be seen that the current of the disconnected bar decreases to zero and the current of the adjacent normal bar increases. Figure 7 shows that after the BRB fault occurs, the end ring current amplitude fluctuates in different amplitudes. In conclusion, the BRB faults lead to a reduction of the current in some rotor bars and end rings, and an increase in the current value in others. However, the reduction of copper loss caused by the broken bars is larger than the increase in copper loss caused by the increase in current. This means that the total rotor copper loss shows a downward trend. This reduction trend becomes less apparent with an increasing number of broken bars.

### 3.3. Iron Loss

The classical iron loss calculation model is a trinomial constant coefficient model [45]. It was proposed by Bertotti, and it divides iron loss into hysteresis loss, classical loss and excess loss. However, this model only studies the influence of alternating magnetic fields on iron loss. This means that it is only applicable to situations of sinusoidal magnetic flux density. Therefore, in order to adapt the model to an IM with broken bars, an improved calculation model that fully considers harmonic, alternating magnetic field and rotating magnetic field has been derived. It is based on the Bertotti trinomial constant coefficient model of iron loss.

The time-domain waveform of the magnetic flux density at each grid element of the iron core is obtained using the finite element method. Here, the irregular elliptical rotating magnetic field with magnetic density is decomposed into two mutually orthogonal alternating magnetic fields. Then the regular elliptical rotating magnetic fields corresponding to fundamental, harmonic and fractional harmonic waves are obtained through the use of harmonic analysis. Total iron loss is equal to the sum of the iron loss generated by the fundamental and each harmonic magnetic density of the two alternating magnetic fields. The specific calculation formulas are as follows:

(4) $$p_{hk}=k_{h}\sum_{k}f_{k}\left(B_{k\max}^{2}+B_{k\min}^{2}\right)$$

(5) $$p_{ck}=k_{c}\sum_{k}f_{k}^{2}\left(B_{k\max}^{2}+B_{k\min}^{2}\right)$$

(6) $$p_{ek}=k_{e}\sum_{k}f_{k}^{1.5}\left(B_{k\max}^{1.5}+B_{k\min}^{1.5}\right)$$

(7) $$p_{\mathrm{Fe}k}=p_{hk}+p_{ck}+p_{ek}$$

(8) $$p_{\mathrm{Fe}}=l_{\mathrm{Fe}}\sum_{k}p_{\mathrm{Fe}k}\cdot S_{k}$$

where *P*_Fe*k*_, *P*_h*k*_, *P*_c*k*_ and *P*_e*k*_ are the iron loss, hysteresis loss, classical loss and excess loss per unit volume of the *k*-th unit, respectively, *k*_h_, *k*_c_ and *k*_e_ are the coefficients of the hysteresis loss, classical loss and excess loss, respectively, *f_k_* is the frequency of the magnetic density, and *B_k_*_max_ and *B_k_*_min_ are the flux density amplitudes of the long and short axis of the regular elliptical rotating magnetic field, respectively, *l*_Fe_ is the effective length of the iron core and *S_k_* is the area of the *k*-th core unit.

**Figure 11:**
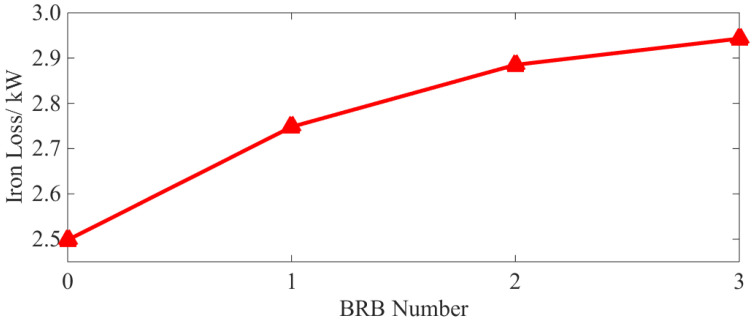
Characteristic curve of iron loss.

Iron loss has been calculated from Equations (4)–(8) under different BRB conditions. This is shown in Figure 11:

According to Figure 11, it can be seen that even with only one broken bar, the iron loss of the squirrel-cage IM is significantly increased. Here, the increase in the iron loss corresponds to the degree of the BRB fault. In addition, the growth rate of iron loss for multiple broken bars is smaller than that for a single broken bar. This is due to the fact that the increase in iron loss comes from the harmonic magnetic field caused by broken bars. More broken bars mean higher harmonic content in the magnetic field. However, due to magnetic field saturation, iron loss increase slows with the increasing number of broken bars.

### 3.4. Mechanical Loss and Additional Losses

Mechanical loss mainly includes bearing friction loss and windage loss. For normal, small and medium-sized squirrel-cage IMs, the mechanical loss is generally calculated according to the following formula [46]:

(9) $$p_{m}={\left(\frac{3}{p}\right)}^{2}D_{1}^{4}\times 10^{4}$$

where *D*_1_ is the outer diameter of the stator.

Additional losses are caused by factors such as pulse vibration of air-gap magnetic flux due to stator and rotor slotting, as well as the harmonics of the stator and the rotor magnetomotive force. These losses are not easy to calculate, but they are usually calculated using the empirical formula in [47]:

(10) $$p_{\mathrm{ad}}=\left(0.025-0.005\log P_{2}\right)P_{1}$$

where *P*_2_ is the mechanical power, *P*_1_ is the electrical power.

### 3.5. Total Loss and Efficiency

The total loss of a squirrel-cage IM can be expressed as:

(11) $$\sum p=p_{\mathrm{sCu}}+p_{\mathrm{rCu}}+p_{\mathrm{Fe}}+p_{m}+p_{\mathrm{ad}}$$

The operating efficiency of an asynchronous motor is expressed as:

(12) $$\eta =\frac{P_{2}}{P_{1}}=\frac{P_{2}}{P_{2}+\sum p}=1-\frac{\sum p}{P_{1}}$$

**Figure 12:**
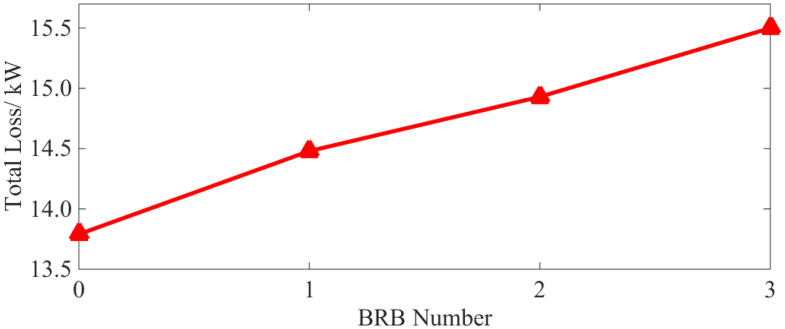
Characteristic curve of total loss.

**Figure 13:**
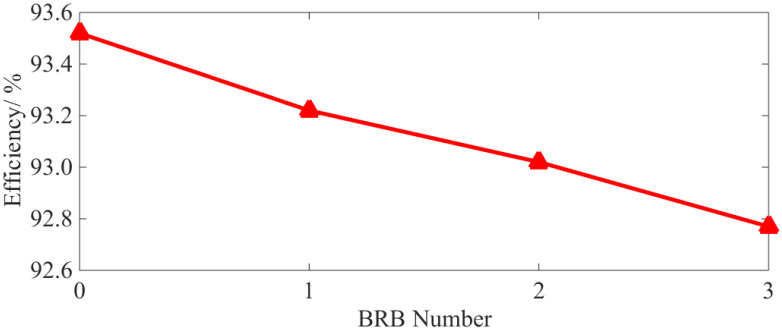
Characteristic curve of efficiency.

According to Equations (11) and (12), the characteristic curves of total loss and efficiency under different BRB fault conditions are provided in Figure 12 and Figure 13, respectively.

As can be seen from Figure 12 and Figure 13, even with only a single broken bar, the increases in the total loss and decreases in the operating efficiency are still clearly visible. Moreover, as the number of broken bars increases, they become even more significant.

## 4. Experimental Verification

### 4.1. Experimental Platform

**Figure 14:**
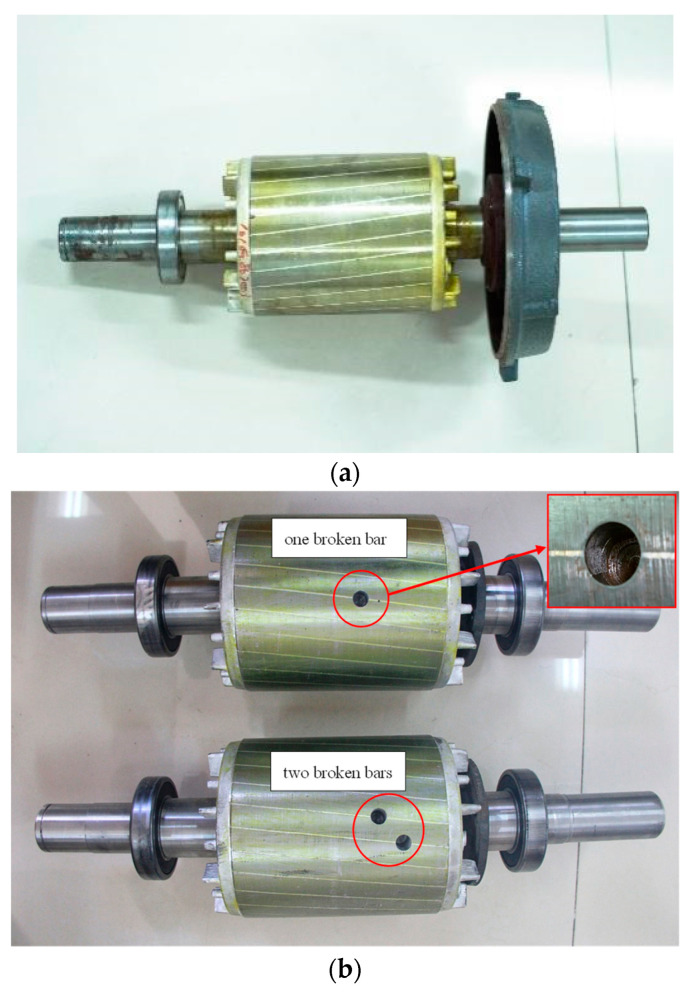
Experimental rotors:(**a**) healthy rotor; (**b**) rotor with broken bars.

**Figure 15:**
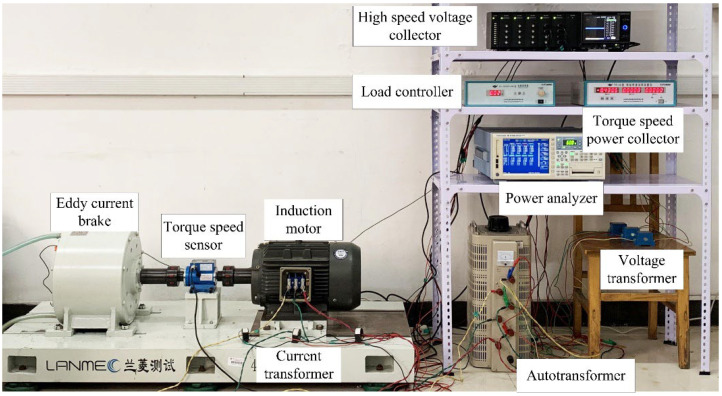
Efficiency test experimental platform.

As the actual high-capacity motors do not have the experimental conditions, a VT132M-4 squirrel-cage IM produced by Hebei Electric Machinery Factory is used for testing and its parameters are shown in Table 2. The rotor has a skewed slot structure with a slot width of 6.9 mm and a slot depth of 21 mm. In order to simulate the BRB fault, a hole is drilled at the rotor bar with a diameter of 10 mm and a depth of 25 mm. Figure 14a,b shows a healthy rotor, a rotor with one broken bar and a rotor with two broken bars, respectively. The efficiency test platform is presented in Figure 15.

The experimental method is implemented according to method B in GB-T 1032-2012 [47] and IEEE 112B [48]. The loss analysis method for measuring input and output power is divided into two parts: the rated-load experiment (conducted first) and the no-load experiment (conducted later) to solve the internal losses and operating efficiency of the squirrel-cage IM.

### 4.2. Experimental Results

In the rated-load experiment, under normal and different BRB fault conditions, the eddy current brake is adjusted through the load controller to keep the squirrel-cage IM running with a rated load. At this time, the torque, speed and output power are all measured in real-time using the torque-speed power collector, as shown in Table 3. Among these, the load torque is displayed as a negative value.

As can be seen from Table 3, the load torque of the squirrel-cage IM remains largely constant. With broken rotor bars, the speed and output power of the squirrel-cage IM decreases with an increase in the number of broken bars.

**Figure 16:**
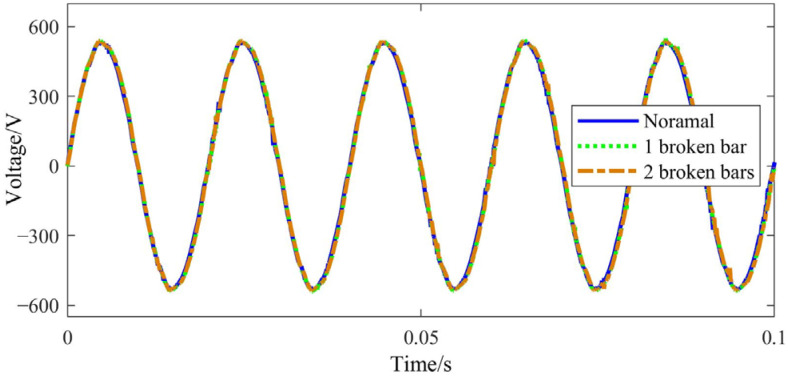
Stator voltage time-domain signal.

**Figure 17:**
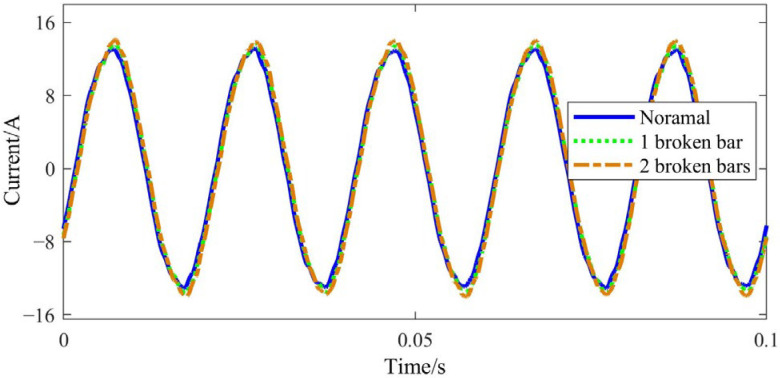
Stator current time-domain signal.

**Figure 18:**
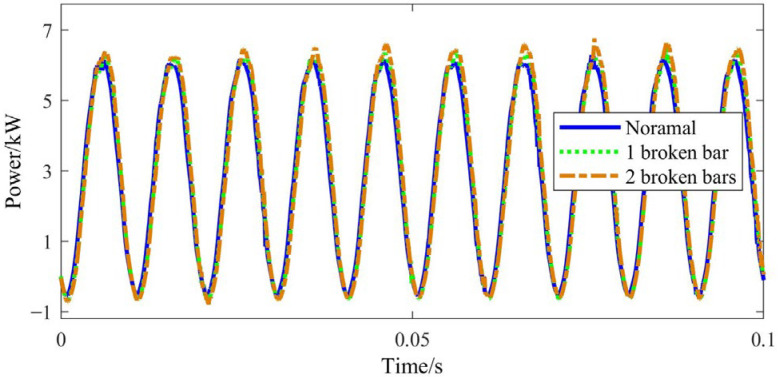
Input power time-domain signal.

Taking phase A as an example, the stator voltage, current and input power waveforms measured in the experiments are provided in Figure 16, Figure 17 and Figure 18, respectively.

It can be seen that both the stator current and input power increase due to the BRB fault, and the input power amplitude shows a slight fluctuation. As the number of broken bars increases, the above phenomenon becomes even more obvious. This is consistent with Figure 3 obtained by theoretical simulation.

**Figure 19:**
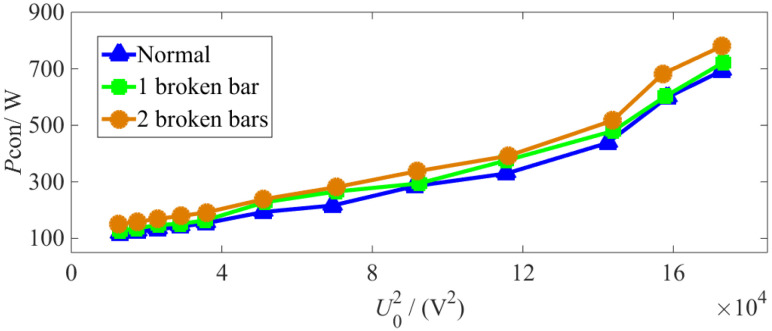
Characteristic curve under no-load conditions.

**Figure 20:**
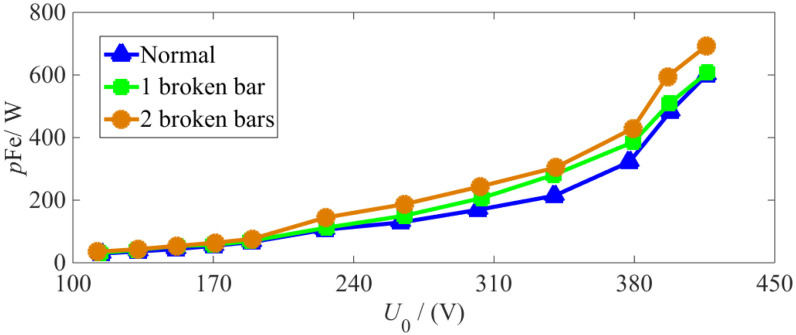
Characteristic curve of iron loss under no-load conditions.

In the no-load experiments, the no-load characteristic curves and iron loss no-load characteristic curves are shown under normal and various BRB fault conditions. (see Figure 19 and Figure 20, respectively).

From Figure 19 and Figure 20, it can be seen that the constant loss and iron loss increase with no-load voltage. When the IM experiences a BRB fault, the constant loss and iron loss increase alongside deterioration of the fault under different no-load voltages.

Once affected by stator and leakage reactance, the induced potential will produce significant errors compared with rated voltage. In order to accurately measure the basic iron loss at a rated load, the curve is obtained according to the induced potential *E* = *U*_0_:

(13) $$E=\sqrt{{\left(U_{N}-I_{P}R_{P}\cos\varphi \right)}^{2}+{\left(I_{P}R_{P}\sin\varphi \right)}^{2}}$$

where *U*_N_ and *I*_P_ are the stator line voltage and phase current at a rated load and voltage, respectively. $\cos\varphi =P_{1}/\left(3U_{N}I_{p}\right)$.

The electromagnetic power PM is transmitted to the rotor by the stator through the law of electromagnetic induction. Here, the s*P*_M_ component becomes the rotor copper loss and the remaining (1 − s)*P*_M_ is converted into mechanical power. Therefore, rotor copper loss *P*_rCu_ can be given as:

(14) $$p_{\mathrm{rCu}}=\left(P_{1}-p_{\mathrm{sCu}}-p_{\mathrm{Fe}}\right)\cdot s$$

**Figure 21:**
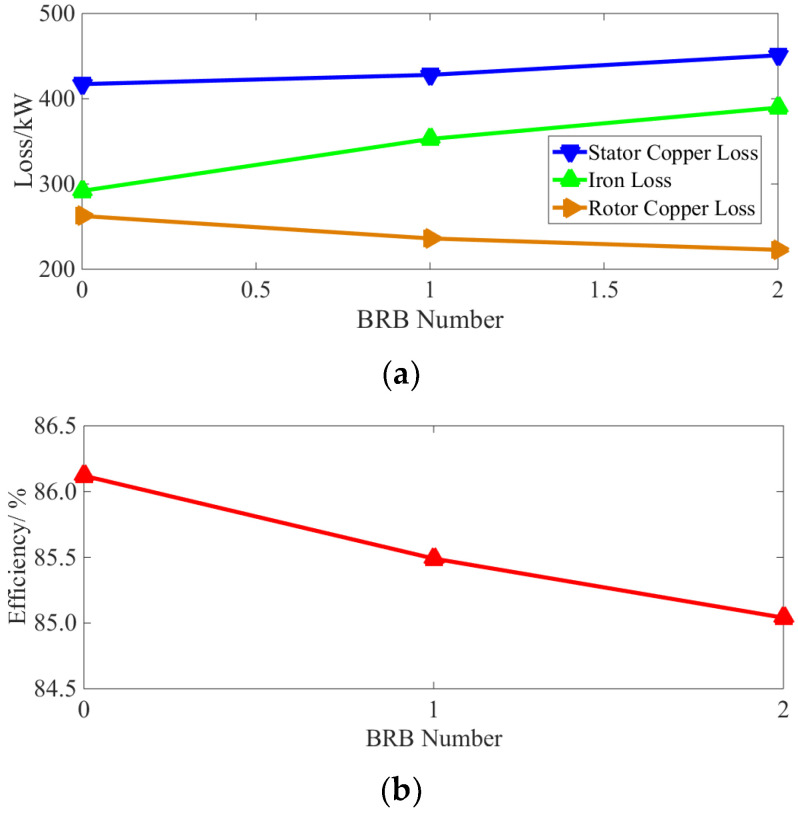
Extra electricity charges during the life period of the unit: (**a**) loss; (**b**) efficiency.

Thus, the stator copper loss can be obtained by substituting the experimental steady-state stator three-phase current into Equation (1), and the iron loss at a rated load is obtained using Equation (13) from the characteristic curve of iron loss under no-load conditions shown in Figure 20. Through Equation (14), the rotor copper loss can then be obtained, and from Equation (12), the operation efficiency of the motor can be calculated. The calculation results of loss and efficiency under normal and different BRB fault degrees are compared in Table 4 and Figure 21a,b.

As can be seen from Table 4 and Figure 21, with a BRB fault, the stator copper and iron loss increases. Although the rotor copper loss is reduced, the overall operational efficiency of the motor is also reduced. Compared with the normal operation state, and even with only one broken bar, the above losses and efficiency vary by 2.59%, 20.86%, 10.04% and 0.63%, respectively. It can be concluded that both the loss increases, and efficiency decreases caused by BRB faults can be serious. They will have adverse effects on motor operation. As the number of broken rotor bars increases, the above phenomenon becomes even more severe, which is consistent with the simulation results (see Figure 9 and Figure 10).

## 5. Strategies to Deal with BRB Fault

To deal with BRB Faults, it is necessary to consider the cost of equipment replacement. Using the same YKK3552-4 squirrel-cage IM as an example, two schemes of replacing equipment and continuing operations are considered. According to the input power, designed annual operation hours and electricity price factors, the annual operation expenses for normal and different degrees of broken bars are obtained. These are shown in Table 5 (It is consistent with the change trend of Table 4 obtained from the experiment). The average electricity price for general industrial and commercial electricity in China is considered to be 0.0910 USD/kWh. In addition, the designed annual operating hours for mine IMs are generally considered to be 6000 h, with a designed life of 15 years. Among them, Loss cost (in Table 5) = *q* broken bars Cost-Normal Cost (0 broken bars Cost). (where *q* is the number of broken rotor bars)

**Figure 22:**
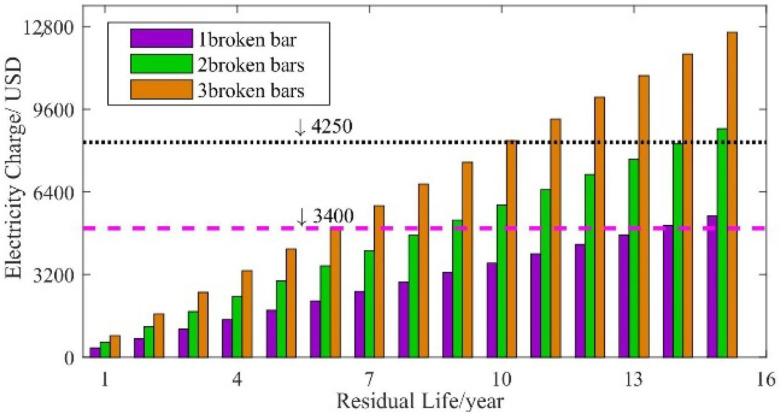
Extra electricity charges during the life period of the unit.

Assuming that the motor suffers broken rotor bars in different years, and its residual life is 1–15 years, the extra electricity cost over the whole life period of the unit is calculated. The cost of electricity during normal operation of the motor is set as reference values. The result is provided in Figure 22.

As can be seen in Figure 22, when the rotor has broken bars, the extra electricity cost due to the increased loss is proportional to the residual life of the motor. It also increases alongside the degree of the BRB fault. This shows that the additional electricity charge is equivalent to the cost of a new rotor or even a new motor after several years of operations. The following is a strategy for dealing with broken rotor bars under different circumstances from the point of view of economic costs. For now, we make the following assumptions:

(1) The design and service life of the stator, rotor and entire squirrel-cage IM machine are all 15 years.
(2) The rotor has broken bars at the beginning of *N*-th year during the use of the motor.

When a broken rotor strip failure occurs, there are three response strategies:

The first equipment-replacement strategy is whole machine replacement:

Using the average depreciation method, the annual depreciation cost of the motor is *M*/15. At this time, the relationship between the annual extra cost *P* caused by the BRB fault and *M*/15 should be considered when deciding whether to replace the equipment. If *P* > *M*/15, the faulty motor should be replaced immediately, whereas there should be no immediate replacement if *P* < *M*/15.

The second equipment-replacement strategy is the rotor replacement (I):

(1) Assuming only the rotor is replaced, the new rotor will operate together with the original stator for (16 − *N*) years.
(2) After 15 years of combined operation, both the stator and rotor are scrapped.

The relationship between the annual extra cost *P* caused by BRB fault and *M*_r_/(16 − *N*) should be considered when deciding whether to replace the equipment. If *P* > *M*_r_/(16 − *N*), it should be replaced immediately whereas there should be no immediate replacement for *P* < *M*_r_/(16 − *N*).

The third equipment-replacement strategy is rotor replacement (II):

(1) Assuming only the rotor is replaced, it will run together with the original stator for (16 − *N*) years.
(2) After the motor reaches its service life of 15 years, the rotor still has a service life of (*N* − 1) years. The rotor is disassembled and combined with a new stator, and it will continue to exert its value. Then the rotor may be replaced later when its service life expires.
(3) Based on this assumption (2), the economic cost of replacing the rotor can be considered using an annual depreciation cost of *A* = *M*_r_/15.

**Figure 23:**
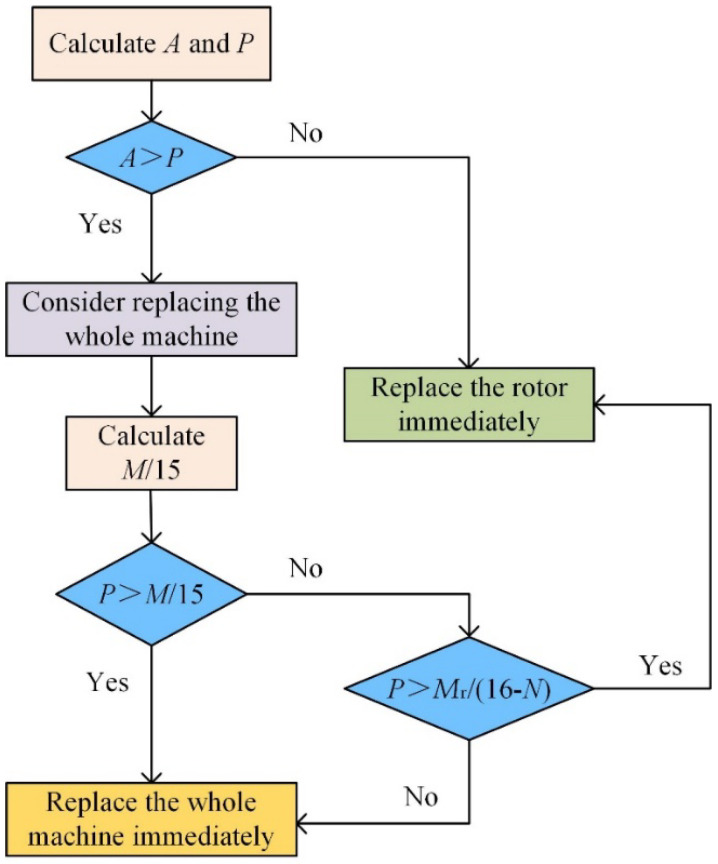
Flow chart of response scheme selection for BRB fault.

Thus, if the value of *A* is greater than the annual extra cost caused by the BRB fault, industrial enterprises should not consider replacing the rotor (provided the rotor can still be used normally, the starting performance changes little, and any vibration during operation is acceptable). Otherwise, the industrial enterprises should replace the rotor immediately. Flow chart for the strategies to deal with a BRB fault in Figure 23.

The meanings of the parameters are as follows:

*A*: the annual depreciation cost of rotor replacement;

*P*: the annual extra cost for rotor bar faults;

*M*: the depreciation cost of new motors;

*M*_r_: the price of new rotor;

The results of the calculations obtained for the above three strategies to deal with BRB fault when faced with different degrees of rotor breakage are shown in Table 6.

The “✗” in Table 6 indicates that the solution is not optimal, the “✓” indicates that the solution is optimal, and a number indicates that this strategy can be used when the remaining life is in that range. For example, “14” means that if an induction motor with a remaining life of 14 years or more fails with one broken bar, strategy 2 can be selected for repair.

**Figure 24:**
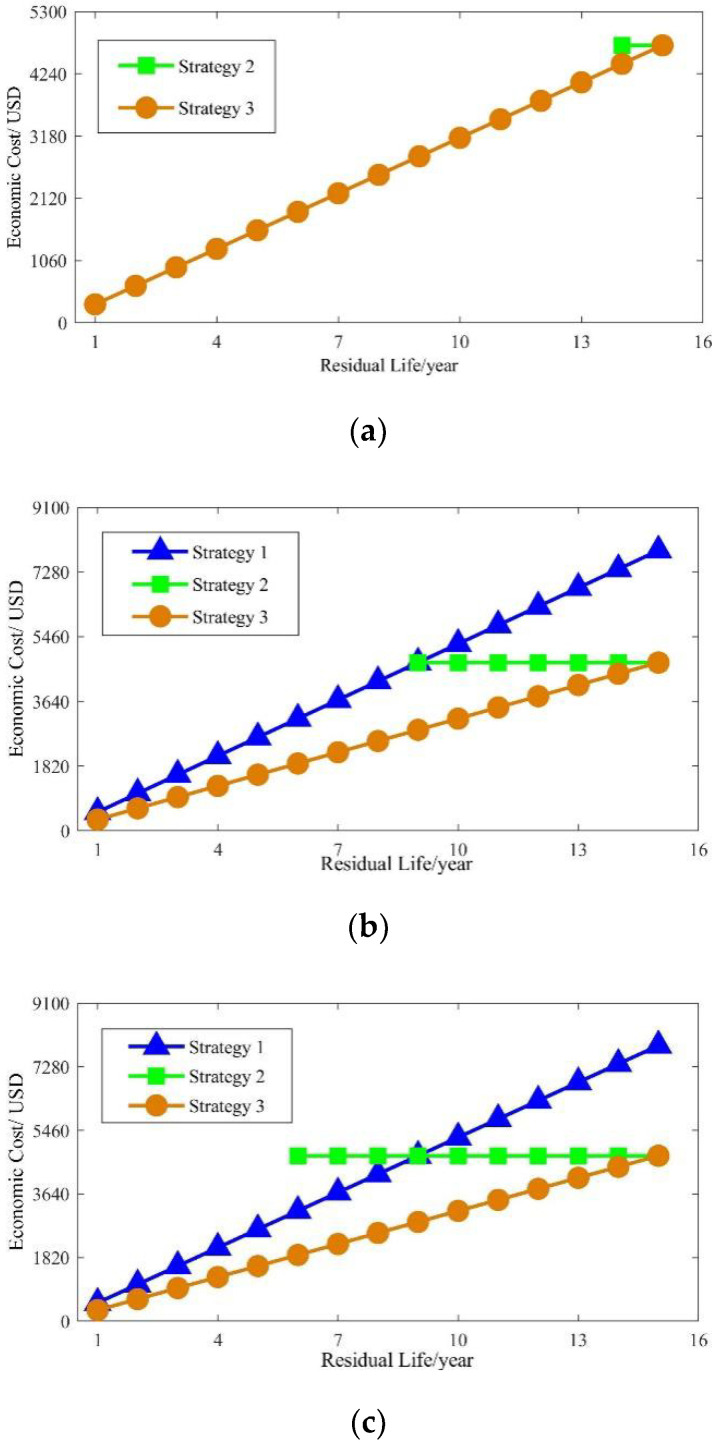
Economic cost of the different equipment-replacement strategies: (**a**) one broken bar; (**b**) two broken bars; (**c**) three broken bars.

In order to achieve the best overall economic benefit, it is necessary to compare the economic costs of the three replacement strategies at different degrees of BRB faults. The calculated cost is presented in Figure 24.

According to Figure 24, when there is only one broken bar, it is not economical to replace the entire machine (strategy 1). This is because the electricity costs due to extra losses over the life period are low. At the same time, the strategy of replacing but not depreciating the rotor (strategy 2) is only applicable to motors with a longer residual life period. However, with the increasing number of broken rotor bars, the extra waste in electric charge over the whole life of the motor is very significant. Therefore, the strategy of replacing but not depreciating the rotor gradually becomes more widely applicable, and eventually, it becomes economical to adopt the strategy of replacing the entire machine. In addition, when facing different degrees of BRB faults, the strategy of replacing and depreciating the rotor always has the lowest cost at different residual life periods of the motor.

## 6. Conclusions

For the first time, this paper focuses on fault treatment strategies for induction motors with broken rotor bars. The influence of BRB faults on the loss and efficiency characteristics of squirrel-cage IMs is analyzed. Through the calculation and evaluation of relevant economic data involved with three replacement strategies, the best path for dealing with different degrees of BRB faults is determined. The following conclusions are obtained:

(1) With a BRB fault, the starting time of the squirrel-cage IM is prolonged. When the number of broken bars is small (1–3 bars), the normal starting of the motor is largely unaffected. In other words, the starting current, starting torque and starting time are all within acceptable ranges (One broken bar has almost no effect on the start-up time. When two bars are broken, the start-up time is prolonged by +5%, and the start-up time for three broken bars is prolonged by +10%). Concurrently, the steady-state speed does not change significantly.

(2) BRB faults will lead to increases of stator copper (+1.4%, +4.65%, +9%) and iron loss (+9.68%, +15.2%, +17.60%) and the decrease in rotor copper loss (−5.04%, −5.94%, −6.89%). Total motor loss (+4.72%, +7.7%, +12.56%) is increased leading to lower operation efficiency (−0.31%, −0.52%, −0.81%). The number of broken rotor bars is positively correlated with the loss of squirrel-cage IM, but negatively correlated with motor efficiency. The decrease in rotor losses as the number of broken rotor bars increases, the larger the number of broken rotor bars, the more obvious this phenomenon becomes.

(3) When facing different degrees of BRB faults, the most appropriate time for maintenance can be determined by comparing the economic costs. In general, when the number of broken strips of the IM is less than 3 and the motor life is long, the strategy of rotor replacement can be used. If the IM has a short remaining life, the user should preferably choose to depreciate the rotor. In conclusion, the strategy of replacing and depreciating the rotor always has the lowest economic cost at different residual life periods of the squirrel-cage IMs, so it should be taken as a suitable strategy for dealing with BRB faults. The results also show that it is necessary to determine the specific number of broken bars during the fault diagnosis stage. We hope that the strategies provided in this paper can be combined with troubleshooting equipment to give users scientific strategies to deal with BRB faults. This is something that requires further research.

From the perspective of ordinary industrial users, this paper provides a new idea to deal with the BRB faults in asynchronous motors. For future work, an intelligent monitoring platform can be developed to minimize losses, reduce costs, improve efficiency and provide scientific maintenance solutions for different asynchronous motors in real industrial environments based on the coping strategies provided in this paper for BRB failures.

## Figures and Tables

**Table 1:** Basic parameters of YKK3552-4 squirrel-cage IM.

Parameter	Value	Parameter	Value
Rated power/kW	200	Number of stator slots	48
Rated voltage/kV	6	Number of rotor slots	34
Power frequency/Hz	50	Rated slip	0.012
Rated power factor	0.85	Core length/mm	320
Rated efficiency/%	94.2	Air gap length/mm	1.4
Number of poles	4	Silicon steel grade	DW470-50

**Table 2:** Nameplate parameters of the experimental squirrel-cage IM.

Parameter	Value	Parameter	Value
Rated power/kW	7.5	Power frequency/Hz	50
Rated voltage/V	380	Rated power factor	0.85
Rated current/A	15.4	Rated efficiency/%	87.0
Rated speed/rpm	1450	Winding connection	Δ

**Table 3:** Output parameters of the experimental motor.

Working Condition	Torque/(N·m)	Speed/rpm	Power/kW
Normal	−49.446	1450.1	7.509
One broken bar	−49.464	1448.0	7.500
Two broken bars	−49.451	1444.5	7.480

**Table 4:** Loss and efficiency of broken bars at different degrees.

Parameter	Normal	One Broken Bar	Two Broken Bars
Stator copper loss/W	417.14	427.93	450.95
Iron loss/W	291.91	352.81	389.57
Rotor copper loss/W	262.51	236.16	222.78
Mechanical loss/W	87.37	94.02	115.31
Additional loss/W	150.92	162.33	169.77
Total loss/W	1209.85	1273.25	1348.38
Efficiency/%	86.12	85.49	84.75

**Table 5:** Annual operating costs with BRB faults at different degrees.

Parameter	Normal	One Broken Bar	Two Broken Bars	Three Broken Bars
Efficiency/%	93.52	93.22	93.01	92.77
Input power/kW	212.94	213.57	213.96	214.39
Cost/k$	116.27	116.61	116.82	117.06
Loss cost/k$	0.00	0.34	0.55	0.85

**Table 6:** Optimal choice of three equipment replacement strategies.

Working Conditions	Strategy 1	Strategy 2	Strategy 3
One broken bar	✗	14	✓
Two broken bars	1–3	✗	✓
Three broken bars	1–3	✗	✓

## Data Availability

Exclude this statement.
